# Analysis of Bacteriophages with Insulator-Based Dielectrophoresis

**DOI:** 10.3390/mi10070450

**Published:** 2019-07-04

**Authors:** Adriana Coll De Peña, Nurul Humaira Mohd Redzuan, Milky K. Abajorga, Nicole Hill, Julie A. Thomas, Blanca H. Lapizco-Encinas

**Affiliations:** 1 Microscale Bioseparations Laboratory and Biomedical Engineering Department, Rochester Institute of Technology, Rochester, NY 14623, USA; 2 Thomas H. Gosnell School of Life Sciences, Rochester Institute of Technology, Rochester, NY 14623, USA

**Keywords:** bacteriophage, dielectrophoresis, electric field, electrophoresis, electrokinetics, virus

## Abstract

Bacterial viruses or phages have great potential in the medical and agricultural fields as alternatives to antibiotics to control nuisance populations of pathogenic bacteria. However, current analysis and purification protocols for phages tend to be resource intensive and have numbers of limitations, such as impacting phage viability. The present study explores the potential of employing the electrokinetic technique of insulator-based dielectrophoresis (iDEP) for virus assessment, separation and enrichment. In particular, the application of the parameter “trapping value” (*Tv*) is explored as a standardized iDEP signature for each phage species. The present study includes mathematical modeling with COMSOL Multiphysics and extensive experimentation. Three related, but genetically and structurally distinct, phages were studied: *Salmonella enterica* phage SPN3US, *Pseudomonas aeruginosa* phage ϕKZ and *P. chlororaphis* phage 201ϕ2-1. This is the first iDEP study on bacteriophages with large and complex virions and the results illustrate their virions can be successfully enriched with iDEP systems and still retain infectivity. In addition, our results indicate that characterization of the negative dielectrophoretic response of a phage in terms of *Tv* could be used for predicting individual virus behavior in iDEP systems. The findings reported here can contribute to the establishment of protocols to analyze, purify and/or enrich samples of known and unknown phages.

## 1. Introduction

Bacteriophages, estimated to have a total population of 10^31^, are possibly the most abundant and genetically diverse biological entities on earth [1,2]. Over the past decade, there has been an increase in observations of antibiotic resistance leading to the need for alternative treatments for bacterial infections. Phage therapy possesses great potential to control multi-drug resistant organisms, such as in the medical and agricultural fields [2]. To employ phages safely for such purposes it is important to have an in-depth knowledge of a representative for each phage group. Our research focuses on understanding the biology of unusually large, so-called “giant” phages, with >200 kb dsDNA genomes, such as *Salmonella enterica* phage SPN3US (240 kb). Increasing numbers of phages that share a core set of genes with SPN3US have recently been isolated, most for the goal of using them for phage therapy purposes. Despite their obvious potential for biocontrol applications, in reality little is known about the biology of these phages. For instance, the virions of giant phages related to SPN3US are comprised of many (>70) different proteins ranging in copy numbers from just a few to 1560 copies per virion, a large proportion of which (~80%) have no known specific function. These characteristics have led us to conduct genetic studies on SPN3US as a model for related “giant” phages.

**Figure 1:**
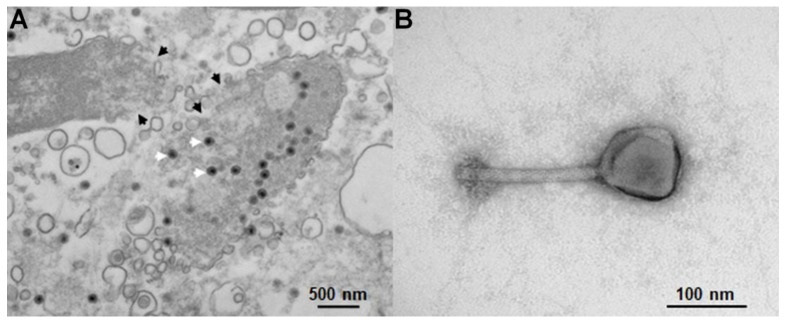
Transmission electron microscopy (TEM) of SPN3US-infected *Salmonella* and purified SPN3US. (**A**) Negatively stained thin section showing two *Salmonella* cells in the process of being lysed at the end of infection by SPN3US. Regions of the cell walls undergoing rupture due to phage enzymes are indicated with black arrowheads. Particles of SPN3US progeny are indicated with white arrowheads. Note the extensive amount of cell debris in the sample. (**B**) Negatively stained image of a single SPN3US virion from a preparation that has undergone purification via CsCl gradient ultracentrifugation to remove cellular debris from the sample. SPN3US virions consist of a head (which contains the dsDNA genome) and tail which ends in a complex baseplate that attaches to a *Salmonella* cell to initiate infection.

To conduct characterization studies on SPN3US, or any phage, requires the use of techniques to purify and enrich particles from a non-homogenous sample containing bacterial cells, cellular debris, and virions, as illustrated in Figure 1. Traditional bacteriophage purification methods, developed for model phages, such as T4 and T7, have numbers of limitations, including being time and labor intensive and often involving a complex series of steps (e.g., CsCl gradient ultracentrifugation) [3]. However, due to the great variability in virion composition between different phages, traditional procedures are frequently not suitable for the purification of many of the newer “environmental” phages, damaging their virions and causing loss of viability. For instance, CsCl gradient purification causes the virions of *Bacillus* virus, phageG to completely disintegrate, and those of *B. thuringiensis* phage 0305ϕ8-36 to lose infectivity by several orders of magnitude [4]. Similarly, even environmental phages that are related to model phages, such as T4, can respond very differently to the protocols employed for the model phage (e.g., [5]). To further complicate matters, standard purification protocols often do not completely remove all bacterial debris, such as cell wall proteins and endotoxins, which can impede downstream analyses (e.g., mass spectrometry) and represents a major problem for therapeutic preparations of phages [6,7]. Given these limitations of traditional phage purification techniques, alternative separation and enrichment processes are being explored [6,7,8].

Microfluidics has revolutionized the manner in which many bioanalytical assessments are performed. It has opened the doors to perform high resolution and sensitivity purification assays [9]. Electrokinetics (EK), electric field-driven techniques, is one of the main pillars of microfluidics due to its great flexibility and simplicity of application. Dielectrophoresis (DEP) has proven to be a robust platform for the separation, sorting and enrichment of a wide array of biological particles ranging from macromolecules to parasites [10,11,12,13,14]. Dielectrophoresis is the migration of particles under the influence of a non-uniform electric field. Unlike electrophoresis (EP), DEP exploits particle polarization effects, not the electrical charge, leading to a greater flexibility since it works with both DC and AC electric potentials [15]. Insulator-based DEP (iDEP) is a technique where non-uniform electric fields are produced employing insulating structures, usually embedded in a microchannel, creating a truly 3-dimensional dielectrophoretic effect [16]. Is it important to note that iDEP systems can suffer from electrolysis and Joule heating effects [17] due to the requirement of high voltages. Therefore, operating conditions need to be carefully selected.

Microorganisms have been extensively studied in dielectrophoretic-based systems, including both electrode-based DEP (eDEP) and iDEP systems [18]. A challenge in the dielectrophoretic manipulation of viral particles is the inherently small size as larger applied electric potentials are required to generate sufficient dielectrophoretic forces [19]. Some of the first studies were focused on the assessment of viruses that are pathogenic to humans. In 1996 the Furh research group demonstrated the enrichment and stable trapping of influenza and Sendai viruses in an eDEP system with two sets of planar electrodes that allowed for the creation of 3D field cages [20,21]. This work was later extended by Grom et al. demonstrating the ability to transport and accumulate hepatitis A virus in a field cage consisting of eight microelectrodes. [19]. Hughes et al. reported a series of studies on the characterization of herpes simplex virus with DEP [22,23,24]. Akin et al. reported an iDEP system with an interdigitated electrode array for real-time trapping and imaging of vaccinia virus [25]. Masuda et al. presented a 3-dimensional iDEP system that allowed the filtration and selective transportation of a single influenza to promote single-virus cell infection [8]. Prakash et al. employed a droplet-based system for the detection of influenza viruses using PCR; illustrating the potential of DEP for diagnostics [26]. Ding et al. utilized gradient iDEP to concentrate Sindbis virus to increase the concentration of the virus from two to six times within the channel using voltages as low as 70 V [12]. Other recent reports have focused on the development of sensors. Singh et al. created a sensor for influenza virus employing carbon nanotubes that were electrodeposited by means of DEP [27]. Madiyar et al. reported the capture and detection of vaccinia virus with DEP by using carbon nanoelectrode arrays [28]. Some earlier studies involved plant viruses. Morgan and Green demonstrated the first application of eDEP for the manipulation of tobacco mosaic virus (TMV) using AC electric fields [29]. Ermolina et al. characterized the dielectric properties of cow pea mosaic virus and TMV in a system with castellated electrodes [30,31]. Lapizco-Encinas reported the enrichment of TMV in an iDEP system with cylindrical insulating posts [32].

In contrast to human and plant viruses, there have been few studies on the suitability of DEP for phage enrichment. Sonnenberg et al developed an eDEP system for the isolation detection of T7 bacteriophage from whole blood [33], illustrating the potential of DEP for clinical applications. Madiyar et al. demonstrated single virus and large ensemble trapping of T4r and T1 bacteriophages from a dilute solution under conditions with a nanoelectrode array made of carbon nanofibers [34]. 

The contributions mentioned above are excellent examples of some of the latest advancements in the dielectrophoretic manipulation of viral particles. However, it is evident that systems capable of purifying newer “environmental” phages, including giant phages, are still an unexplored area. Similarly, unexplored are systems capable of handling a larger throughput containing several viral species which would be of potential value in phage therapy as typically cocktails or mixtures of different types of phages are employed. In this contribution, we present the first report on the assessment and enrichment of *Salmonella* phage SPN3US, and for comparison purposes, two related giant *Pseudomonas* phages: ϕKZ and 201ϕ2-1, in two distinct iDEP systems. In particular, the application of the parameter “trapping value” (*Tv*) is explored as a standardized iDEP signature for each virus species. This work includes mathematical modeling with COMSOL Multiphysics® (version 4.4, COMSOL Inc., Stockholm, Sweden) and experimentation with iDEP devices containing an array of circular or oval-shaped insulating posts. For model information, please see the supplementary material. The dielectrophoretic trapping of viral particles under the influence of DC electric potentials was fully characterized in order to discern the specific trapping conditions (“sufficient” trapping) for each one of the distinct viral species. All viruses in this study exhibited negative dielectrophoretic behavior. The results illustrating virus trapping and enriching allowed the identification of the specific Trapping value (*Tv*, Equation (3)) for each type of phage [35,36]. This is the first iDEP study on large bacteriophages and these findings could be used for the design of new iDEP systems aimed to separate and enrich samples of both known and unknown phages. 

## 2. Theory

**Figure 2:**
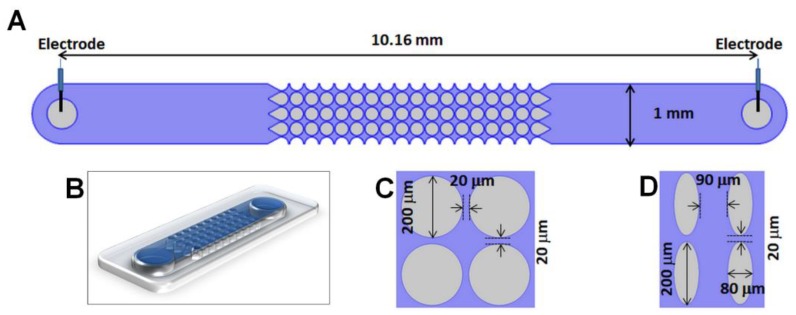
Schematic representation of one insulator-based dielectrophoresis (iDEP) channel employed in this study. (**A**) Top view of a full channel for design Circle-200-220. (**B**) 3D representation of the channel. For the two designs analyzed in this study, an illustration of four insulating posts with dimensions is included: (**C**) Circle-200-220, (**D**) Oval-200-220&80-170. Design names illustrate post size and post spacing.

Particles can exhibit either positive or negative DEP, depending on their relative polarizability with respect to the suspending media [37]. Positive DEP (pDEP) occurs when the particle is more polarizable than the medium, resulting in particle attraction to the regions with higher electric field gradient. Negative DEP (nDEP) is the opposite effect. In our iDEP channels (Figure 2A,B), the constrictions between posts are the areas of high field gradients. Under nDEP, all virus species in this study could be trapped in the constriction regions. In iDEP systems particles are captured when the effects of DEP and linear EK forces, which are opposite, are balanced [38]. For a particle to become trapped the following condition has been identified [39,40]:

(1) $$\frac{\mu_{DEP}\nabla E^{2\;}\cdot \vec{E}}{\mu_{EK}\;E^{⃑}\cdot \vec{E}}\leq -1,$$

Separating the above expression into system-dependent and particle-dependent parameters:

(2) $$\frac{\nabla E^{2\;}\cdot \vec{E}}{E^{2\;}}\leq -\frac{\mu_{EK}}{\mu_{DEP}},$$

where the left-hand side of the equation is the Trapping value which is independent of particle properties, as it only depends on the electric field magnitude ($\vec{E}$) and gradient of the electric field squared ($\nabla E^{2}$). This parameter, identified by the Casals-Terré [35] and Hayes [36] groups, characterizes the condition required to trap a specific type of particle:

(3) $$Tv=\frac{\nabla E^{2\;}\cdot \vec{E}}{E^{2\;}}.$$

## 3. Materials and Methods

### 3.1. Microdevices, Viral Samples and Suspending Medium

Experiments were conducted in two distinct microchannel designs made from PDMS employing standard soft lithography techniques; microfabrication information is included here [40]. The microchannels were 10.16 mm long, 40 µm deep and 880 μm wide, specific post dimensions are included in Figure 2C,D. This study employed high titer stocks (10^10^–10^12^ pfu (plaque-forming units)/mL) of three related viruses: *Salmonella* Typhimurium phage SPN3US [41], *Pseudomonas aeruginosa* phage ϕKZ [42] and *P. chlororaphis* phage 201ϕ2-1 [43]. These phage stocks underwent a low speed clarification spin (~8000 g, 10 min, 4 °C) to remove large bacterial debris. All virus samples were fluorescently labeled as follows: 1 mL of a phage stock was spun down at 13,000 rpm for 10 min, after discarding the supernatant the pellet was resuspended in 0.5 mL of distilled water. Then, 2 µL of SYTO 11 dye (Invitrogen, Carlsbad, CA, USA) was added to the sample and incubated for 20 min. After removing the excess dye, the sample was resuspended in 0.5 mL of the suspending medium. The suspending medium was sterilized deionized water with a conductivity of 14 μS/cm and a pH of 7.07; under these conditions the zeta potential of the PDMS channel was approximately −108.57 mV.

Phage samples were assayed for viability via plaque assays in triplicate using the standard double overlay technique using LB agar bottom plates and overlays made from LB broth and 0.34% agar. Briefly, each phage sample underwent a 10-fold dilution series in SM buffer and these were spotted onto overlays made containing 100 μL of a fresh overnight culture of the appropriate bacterial strain. Plaques were enumerated after overnight incubation at 30 °C.

### 3.2. Equipment and Experimental Procedure

Phage response was observed and recorded as videos with a Leica DMi8 inverted microscope (Wetzlar, Germany). Direct current (DC) electric potentials were applied with a high voltage supply (Model HVS6000D, LabSmith, Livermore, CA, USA). COMSOL Multiphysics® 4.4 was used to predict the magnitude of the trapping value (*Tv*, Equation (3)). Each experiment started with a clean channel to which a 5–10 μL sample of the corresponding labeled virus was added, followed by the application of DC electric potentials. For the purpose of this study, a “sufficient” trapping voltage was determined as the required voltage to obtain a visually observable band or cluster of trapped viral particles. 

## 4. Results and Discussion

### 4.1. Experimental Characterization of the Dielectrophoretic Trapping of Phage Virions

**Figure 3:**
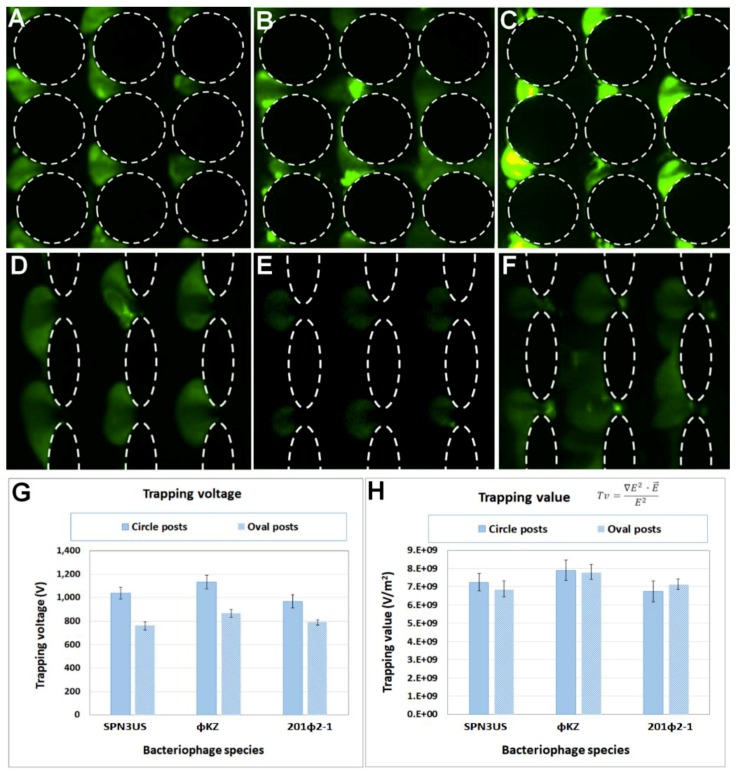
Results of the dielectrophoretic trapping of all three phages. Circle-shaped posts: (**A**) SPN3US at 1200 V, (**B**) ϕKZ at 1100 V and (**C**) 201ϕ2-1 at 1100 V. Oval-shaped posts: (**D**) SPN3US at 800 V, (**E**) ϕKZ at 750 V and (**F**) 201ϕ2-1 at 750 V. (**G**) Experimental characterization of the trapping voltage, and (**H**) Estimation of the trapping value (*Tv*) in both iDEP channel designs for the three types of bacteriophages in this study. Table S1 in the Supplementary Material includes a summary of the trapping voltage and *Tv* estimations.

A series of experiments were carried out to characterize the required voltage to trap and enrich each type of phages in both iDEP devices with nDEP. After a sample of the fluorescently labelled virus was introduced into the channel, the applied voltage was manually increased until “sufficient” trapping of the viral species was observed. Each experiment was repeated at least five times to ensure reproducibility, a summary of these results is included in Table S1 (supplementary material). Figure 3A,C illustrate images of the trapping of all three phage species in the circle-shaped iDEP channel at applied potentials between 1100 and 1200 V. Lower voltages, in the range of 750–800 V, were required with the oval-shaped posts, as depicted in Figure 3D,F. Figure 3G shows a plot of the required trapping voltage necessary to achieve “sufficient” trapping. As expected, for all viral species, the required voltages are lower with the oval-shaped posts, since narrower posts generate higher electric field gradients ($\nabla E^{2}$), producing greater dielectrophoretic forces [40]. The characteristic trapping voltage and *Tv* for each viral species is a strong function of the size, shape and polarizability of the viral species. As demonstrated by Hughes et al. [44] the total conductivity of a particle depends on the conductivity of the bulk material, and the individual conductances of the compact and diffuse layers of the electrical double layer (EDL). This group successfully extended this analysis with the dielectrophoretic characterization of simplex virus-1 capsids [45]. In a later contribution, Ermolina et al. [30] illustrated that surface conductance, which is directly related to polarizability, is a dominant parameter in the EP response of submicron particles, such as viruses. 

### 4.2. Modeling Predictions for the Trapping of Phage Virus

The trapping value (*Tv*), which characterizes the conditions required to trap a specific type of particle [35,36], was determined using COMSOL Multiphysics® software (Table S1). The geometries of interest were imported into COMSOL along with trapping voltage (Figure 3G) associated for each species in order to predict the parameters $\nabla E^{2}$, $\vec{E}$ and $E^{2}$. These values were estimated across a cutline located at the centerline of one constriction between two posts. Images depicting the cutlines used in these estimations are illustrated in Figure S1.

As defined by Casals-Terré [35] and Hayes [36] groups, the *Tv* parameter normalizes the required conditions for trapping a specific type of particles for any type of iDEP design. The results in Figure 3H confirm the applicability of *Tv*. It can be observed for each one of the three viral species that the *Tv* values for both iDEP designs are quite similar, a finding which is consistent with our previous analyses that each of these three phages has a large virion composed of mostly homologous proteins and very similar dimensions [46]. Notably, the trapping voltage (Figure 3G) for each phage was unique, which indicates that if, in future studies, the separation of a mixture of similarly related viruses was required, we should focus on the trapping voltages, not the trapping values. In addition, our results indicate that devices with wider posts might be more suitable for such separation purposes (i.e., wider posts (circles) produced trapping voltages with a larger distribution between the three virus species than obtained with narrow posts (ovals)). Furthermore, these findings open the exciting possibility of using *Tv* for the designing of iDEP devices with distinct post geometries; and also for predicting the trapping conditions of different types of particles (from viruses to cells) in a given iDEP device. Consequently, these findings will be relevant for future studies on mixtures of phages, even those including related phages, as found in phage therapy cocktails.

### 4.3. Viability Assessments after Dielectrophoretic Trapping

**Figure 4:**
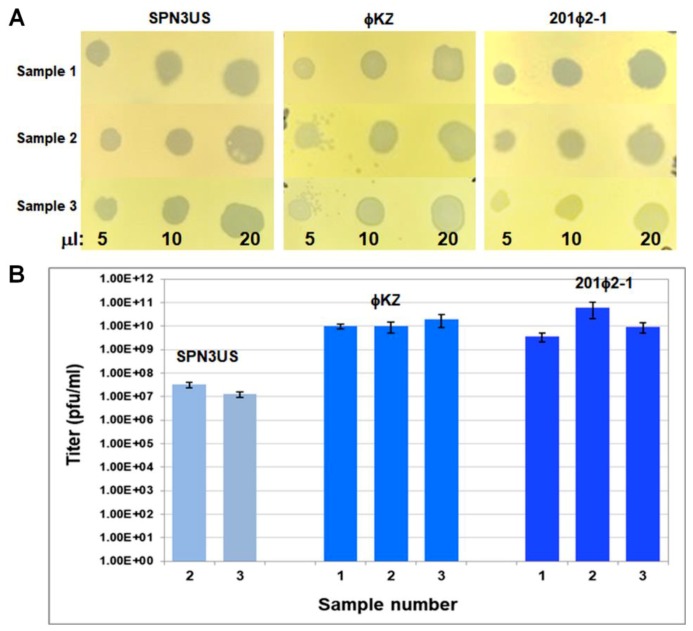
Qualitative viability assessments for all three phages studied here. (**A**) Three samples of phages SPN3US, ϕKZ and 201ϕ2-1 that had been fluorescently labelled and treated were spotted onto the lawns of their respective bacterial hosts in three replicate experiments. Volumes of phage spotted are indicated at the bottom of the image. (**B**) Enumeration of viable particles (plaque-forming units, pfu) of the samples in (A) for phages SPN3US, ϕKZ and 201ϕ2-1, with the exception of SPN3US sample 1 which was not able to be titered.

To evaluate the potential of iDEP for bacteriophage purification and enrichment, the phage samples were assessed for viability after exposing them to high voltages. To do this, the stained input samples that had been previously fluorescently stained for the trapping voltage experiments were run on a circle post design in triplicates, and exposed to the following sequence of voltages: 400 V for 30 s, 800 V for 20 s and 400 V for 10 s. The voltages were chosen to represent the experimental conditions with an initial voltage to move the sample to the post array, followed by a trapping voltage and a release voltage to move the sample to the outlet reservoir prior to extraction. While some sample did reach the outlet reservoir without experiencing the total magnitude of the electric field gradient within the constrictions at 800 V, all viruses retrieved were still exposed to high voltages for at least one minute. Upon extraction of the samples from their respective channels, the samples of each phage were plated on their respective bacterial host. The clearings in the bacterial lawns observed in Figure 4A represent bacterial cell lysis generated by the presence of viable bacteriophages. Remarkably, the two *Pseudomonas* phages, ϕKZ and 201ϕ2-1, had titers in the high range after trapping (Figure 4B, Table S2), a finding which supports that iDEP can indeed be used as a purification technique and not only as an analytical tool for bacteriophages. 

The titers of SPN3US after iDEP were more variable despite comparable trapping magnitudes of its virions in the iDEP channels relative to ϕKZ and 201ϕ2-1 (Figure 4). Potential causes for the SPN3US titer variability include that certain parts of its virion may be more susceptible to damage during trapping after virions have been treated with SYTO 11 than similar structures in ϕKZ and 201ϕ2-1. This seems highly likely as our analyses indicate that each phage has a reduced viability immediately after SYTO 11 treatment (Table S3). It is also feasible that some of the variation in the viability of the SPN3US sample extracted from iDEP may have been the consequence of variation within the channel to the outlet reservoir. Potential future research directions could focus on increasing the yield of enriched phages, and quantification of iDEP relative to existing phage purification techniques with regard the amount of bacterial contaminants removed.

## 5. Conclusions

Presented here is the assessment of three phages: SPN3US, ϕKZ and 201ϕ2-1 in two distinct iDEP devices, one with circle-shaped and one with oval-shaped insulator posts. Experimental work demonstrated the successful trapping of all three phage species, where the voltage requirement to achieve trapping of virions was lower in devices with circular insulating posts, since these produce lower dielectrophoretic forces than the oval-shaped posts. A mathematical model created with COMSOL was then employed to estimate the trapping value (*Tv*) for each phage type. This parameter, as identified by other research groups, normalizes the required conditions, in terms of electric field and electric field gradient, for trapping a specific type of particle. The results demonstrated that the *Tv* for a specific species is reasonably constant within the two distinct designs studied here, opening the exciting possibility of using *Tv* for the designing of iDEP devices targeting specific viral species, and also for predicting the required voltage for trapping a specific type of particle, including viruses, in distinct iDEP devices. In addition, these findings suggest that iDEP has potential for analyses of mixtures of phages, even those including related phages, such as found in phage therapy cocktails.

## Supplementary Materials

The following are available online at https://www.mdpi.com/2072-666X/10/7/450/s1. Figure S1: Representation of the cutline employed in COMSOL for the determination of *Tv*. Table S1: Comparison between trapping voltages and *Tv* of SPN3US, ϕKZ, and 201ϕ2-1 in the circle and oval post channel designs. Table S2: Enumeration of viable particles of phages SPN3US, ϕKZ, and 201ϕ2-1 after trapping in the circle designs. Table S3: Enumeration of viable particles of phages SPN3US, ϕKZ, and 201ϕ2-1 before and immediately after staining with SYTO 11.
