# Supplementary material for: Analysis of Bacteriophages with Insulator-Based Dielectrophoresis
Source: Micromachines (Basel). 2019 Jul 4;10(7):450. doi: 10.3390/mi10070450 (PMC6680707; doi:10.3390/mi10070450)
Supplement: Supplementary file 1 [file micromachines-10-00450-s001.pdf]

Supplementary information: Analysis of bacteriophages with insulator-based dielectrophoresis

Adriana Coll De Peña [1,2], Nurul Humaira Mohd Redzuan [2], Milky Abajorga [2], Nicole Hill [1] Julie A. Thomas [2,*] and Blanca H. Lapizco-Encinas [1,*]

[1] Microscale Bioseparations Laboratory and Biomedical Engineering Department, Rochester Institute of Technology, Rochester, NY 14623, USA
[2] Thomas H. Gosnell School of Life Sciences, Rochester Institute of Technology, Rochester, NY 14623, USA
Correspondences: jatsbi@rit.edu (J.A.T.); bhlbme@rit.edu (B.H.L.-E.); Tel.: +1-585-475-2375 (J.A.T.); +1-585-475-2773 (B.H.L.-E.).

Mathematical Model

Microchannels containing arrays of circular and oval shaped insulating posts were used in this study. The presence of these insulating posts that transverse the entire depth of the microchannel, distorting the electric field distribution across the channel, creates electric field gradients that result in dielectrophoretic effects. A schematic representation of one of the microchannels is included in Figure 2A. COMSOL *Multiphysics* 4.4 (COMSOL, Inc., Newton, MA, USA) with the AC/DC module was used to estimate the distribution of the electric field and gradient of the electric field. The two model built for this study used a mesh of approximately 260,000 and 1,155,000 elements, for the circles and ovals, respectively, considering a two dimensional model. Suspending medium relative permittivity of 78.4 and medium viscosity of 8.91×10^{-4} kg·m^{-1}·s^{-1} were assumed. A brief description of the model is included below. Laplace equation was employed to describe the distribution of the electric potential in the microchannel:

$$\nabla^2\phi = 0, \tag{S1}$$

where φ is the electric potential. The following boundary conditions were considered:

$$\vec{n}\cdot\vec{J} = 0 \quad \text{at the boundaries,} \tag{S2}$$

$$\phi = V_{inlet} \quad \text{at the inlet of the microchannel,} \tag{S3}$$

$$\phi = V_{outlet} \quad \text{at the outlet of the microchannel,} \tag{S4}$$

where $\vec{n}$ is the normal vector to the surface, $\vec{J}$ is the electrical current density and V_{inlet} and V_{outlet} are the electrical potentials at the microchannel inlet and outlet reservoirs. The boundaries considered are the microchannel walls and the surfaces of the oval shaped insulating posts.

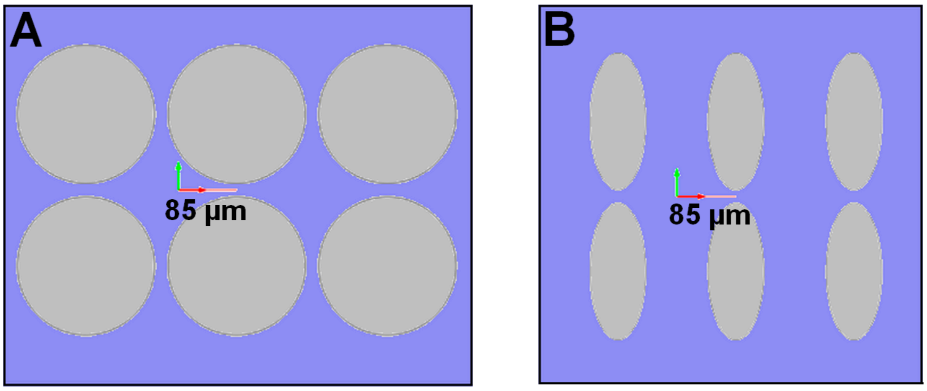

Figure S1. Representation of the cutline employed in COMSOL for the determination of *Tv* (Equation (9)), the cutline in both designs is 85 µm long and illustrated in red color. (A) Circle design and (B) Oval design. This specific cutline length corresponded to half of the constriction

length and half of the horizontal spacing between posts for the posts of the smallest width (Oval-200-220&80-170 design, Figure 2D).

Table S1. Comparison between trapping voltages and values of SPN3US, ϕKZ, and 201ϕ2-1 in the circle and oval post channel designs obtained from experimental data and COMSOL simulations, respectively.

**Parameters/Virus**	**Circle Post**		**Oval Post**	
	**Trapping Voltage (V)**	**Trapping Value, *Tv* (V/m²)**	**Trapping Voltage (V)**	**Trapping Value, *Tv* (V/m²)**
SPN3US	1038 ± 48	3.28E + 09 ± 4.74E + 08	761 ± 35	5.87E + 09 ± 4.5E + 08
ϕKZ	1133 ± 58	3.58E + 09 ± 5.7E + 08	864 ± 32	6.66E + 09 ± 4.1E + 08
201ϕ2-1	967 ± 58	3.05E + 09 ± 5.7E + 08	790 ± 22	6.09E + 09 ± 2.89E + 08

Table S2. Enumeration of viable particles (plaque forming units, pfu) of phages SPN3US, ϕKZ, and 201ϕ2-1 after trapping in the circle designs. Three samples for each phage were enumerated after trapping.

**Samples**	**SPN3US (pfu/mL)**		**ϕKZ (pfu/mL)**			**201ϕ2-1 (pfu/mL)**		
Titers (pfu/ml)	Sample 2	Sample 3	Sample 1	Sample 2	Sample 3	Sample 1	Sample 2	Sample 3
After iDEP R1	2.40E+07	1.60E+07	1.20E+10	4.00E+09	1.20E+10	4.00E+09	1.20E+11	1.60E+10
After iDEP R2	3.60E+07	1.20E+07	8.00E+09	1.60E+10	1.20E+10	2.00E+09	1.00E+11	1.20E+10
After iDEP R3	2.60E+07	1.80E+07	8.00E+09	1.60E+10	1.20E+10	2.00E+09	4.00E+10	6.00E+09
After iDEP R4	4.40E+07	1.00E+07	8.00E+09	8.00E+09	3.60E+10	4.00E+09	1.40E+10	6.00E+09
After iDEP R5	2.60E+07	1.00E+07	1.20E+10	8.00E+09	3.20E+10	4.00E+09	4.60E+10	1.00E+10
After iDEP R6	4.20E+07	1.00E+07	1.20E+10	8.00E+09	1.60E+10	6.00E+09	4.40E+10	6.00E+09
Mean	3.30E+07	1.27E+07	1.00E+10	1.00E+10	2.00E+10	3.67E+09	6.07E+10	9.33E+09
Standard Deviation	8.83E+06	3.50E+06	2.19E+09	4.90E+09	1.10E+10	1.51E+09	4.04E+10	4.13E+09
Standard Error	3.61E+06	1.43E+06	8.94E+08	2.00E+09	4.50E+09	6.15E+08	1.65E+10	1.69E+09

Table S3. Enumeration of viable particles (plaque forming units, pfu) of phages SPN3US, ϕKZ, and 201ϕ2-1 before, and immediately, after staining with SYTO 11.

**Samples**	**SPN3US (pfu/mL)**	**ϕKZ (pfu/mL)**	**201ϕ2-1 (pfu/mL)**
**Original stock - R1**	2.40E+10	2.40E+11	5.20E+12
**Original stock - R2**	2.00E+10	2.20E+11	5.60E+12
**Original stock - R3**	3.60E+10	4.80E+11	5.00E+12
**Original stock - Mean**	2.67E+10	3.13E+11	5.27E+12
**Treated with SYTO 11-R1**	5.60E+09	2.20E+11	6.00E+11
**Treated with SYTO 11-R2**	1.00E+10	2.00E+11	2.00E+11
**Treated with SYTO 11-R3**	1.00E+10	2.80E+11	1.20E+12
**Treated with SYTO 11-Mean**	8.53E+09	2.33E+11	6.67E+11
**Percentage of viable phages remaining after staining**	**16.00 %**	**37.23 %**	**6.33 %**
